# Prerequisites for effective adenovirus mediated gene therapy of colorectal liver metastases in the rat using an intracellular neutralizing antibody fragment to p21-Ras

**DOI:** 10.1038/sj.bjc.6600089

**Published:** 2002-02-01

**Authors:** B van Etten, T L M ten Hagen, M R de Vries, G Ambagtsheer, T Huet, A M M Eggermont

**Affiliations:** University Hospital Rotterdam-Daniel den Hoed Cancer Centre, Department of Surgical Oncology; Room Ee 102, PO Box 1738, 3000 DR Rotterdam, The Netherlands; Aventis-Pharma, Vitry-sur Seine, France

**Keywords:** ras oncogene, gene therapy, adenovirus, liver metastases, hepatic artery infusion, isolated liver perfusion

## Abstract

Ras mutations are present in 40–50% of colorectal cancers. Inactivating this oncogene may therefore reduce proliferation capacity. In order to target ras we studied the transduction efficacy and anti tumour activity of an adenoviral vector expressing an intracellular, neutralizing single chain antibody to p21-ras (Y28). In *in vitro* studies transfection levels of the K-ras mutated rat colon carcinoma cell line CC531 were studied using the LacZ marker gene. In our *in vivo* liver metastases model different routes of administration were evaluated to determine which regimen resulted in the best transfection levels and tumour responses: intravenous injection, intratumoural injection, isolated liver perfusion, or hepatic artery infusion. CC531 cells are readily transfected *in vitro*, resulting in significant inhibition of tumour cell proliferation by the Y28 construct. Intravenous injection did not result in any measurable transfection. Intratumoural injection resulted only in the transfection of tumour cells along the needle track. IHP as well as single HAI achieved low transfection levels of tumour tissue. Expression of Y28 was demonstrated in tumours after IT injection, HAI and IHP. Whereas, repeated HAI's clearly achieved expression in and around tumour associated vessels. Only five times repeated HAI's with Y28 resulted in a tumour response: in all animals tumour growth was inhibited, and in three rats out of eight a complete regression of the liver tumours was observed.

*British Journal of Cancer* (2002) **86**, 436–442. DOI: 10.1038/sj/bjc/6600089 www.bjcancer.com

© 2002 The Cancer Research Campaign

## 

After resection of the primary tumour recurrent colorectal carcinoma occurs in about 50% of patients. In these patients the liver is the major site of metastatic disease (Sugarbaker, 1994). Patients with resectable liver metastases may have a partial hepatectomy with a 5 year survival rate of 25–30% (Hughes, 1988; Steele and Ravikumar, 1989; Scheele *et al*, 1995). On the other hand the natural history of patients with untreated liver metastases shows a 5-year survival rate of 0–3% (Wood *et al*, 1976).

Colorectal carcinogenesis is associated with multiple genetic alterations. Ras mutations occur in an early stage of progression from adenoma to carcinoma. Ras mutations are present in 40–50% of human colorectal tumours (Spandidos *et al*, 1995). Physiologically, the ras gene leads to the production of p21-ras, a protein that catalyses the hydrolysis of guanosine triphosphate to guanosine diphosphate, and in this way controls cell proliferation by regulating signal transduction pathways (Bos, 1989). Inhibition of expression of mutated ras has been shown to cause tumour growth inhibition and apoptosis in human and murine tumour cell lines (Mukhopadhyay *et al*, 1991; Georges *et al*, 1993; Shirasawa *et al*, 1993; Cochet *et al*, 1998, 1999).

In the development of gene therapy protocols it has been often shown that data from *in vitro* experiments do not always predict anti-tumour effects *in vivo*. The main reason for this may be insufficient tumour targeting. By locoregional administration of the genetic construct, tumour targeting may be improved and consequently result in more favourable responses. Previously, we have demonstrated successful transfection and anti-tumour activity in a rat sarcoma model with isolated limb perfusion (de Roos *et al*, 2000; de Wilt *et al*, 2001).

Isolated liver perfusion and hepatic artery infusion are used in surgical oncology trials for the administration of chemotherapeutics and cytokines in patients with liver tumours (Marinelli *et al*, 1990; Marinelli *et al*, 1991; Kemeny *et al*, 1994; Kuppen *et al*, 1997; Alexander *et al*, 1998; de Vries *et al*, 1998). High local drug concentrations can be achieved at the tumour site by means of isolated hepatic perfusion (IHP), a technique with minimal systemic exposure. de Roos *et al* (1997) and others have already demonstrated effective administration of adenoviral vectors via IHP (van der Eb *et al*, 1998a,b).

Repetitive locoregional administration of drugs can be achieved by regional infusions via the hepatic artery (Riemenschneider *et al*, 1988; Miyazaki *et al*, 1993; Kemeny *et al*, 1994). Thus repeated delivery of adenoviral vectors via the hepatic artery may further increase the efficacy of transfection.

We anticipated that the route of delivery plays a crucial role in optimizing transduction efficacy and more importantly in anti-tumour activity. We report here on a transduction efficacy study of an adenoviral vector encoding the LacZ marker gene, administrated to the liver via systemic and different locoregional routes of administration, and subsequently on the antitumour activity of an adenoviral vector expressing a single chain antibody to p21-ras (Y28) *in vitro* and *in vivo* using the rat colon carcinoma CC531.

## MATERIALS AND METHODS

### Recombinant adenovirus constructs

AV1.0CMV.Y28 is a recombinant replication-deficient adenovirus vector expressing the Y28 gene. It encodes the hypervariable regions of an anti-p21-ras single chain antibody driven by the human cytomegalovirus (CMV) promoter. It is derived from the rat Y13-259 monoclonal antibody to p21-ras (Furth *et al*, 1982; Werge *et al*, 1990; Cochet *et al*, 1998). The Y28 expression unit, which also contains the bovine Growth Hormone polyadenylation signal (bGH polyA), replaces the E1 adenovirus region. The AV1.0CMV.Y28 backbone is an E1/E3 deleted human adenovirus serotype 5. This construct was subjected to multiple plaque purification and produced in the 293 cell line (human transformed primary embryonal kidney cell line) trans-complementing for E1 gene products. Adenovirus was recovered from cell culture supernatant and purified by two rounds of cesium chloride ultracentrifugation. Purified virus was then gel-filtered on a PD10 column with a PBS buffer containing 0.5 mM MgCl_2_, 0.5 mM CaCl_2_ and 10% glycerol. Virus stock was aliquoted and stored at −80°C until used. The batch used, met the preclinical grade specifications (Quality Control analyses), in regard to sterility, endotoxins, mycoplasma, viral particles and plaque forming unit titers. AV1.0CMV.LacZ and AV1.0CMV are also recombinant replication-deficient adenovirus vectors constructed on the same basis of an E1 and E3 deleted human adenovirus type 5 backbone and produced in 293 packaging cell line. The former expresses the *E. coli* derived ß-galactosidase protein that can be detected by histochemistry in order to access the transduction efficacy of the vector. The latter contains only the CMV promoter and SV40 signal without any transgene inserted. This ‘empty’ construct has been used as the control vector in all experiments.

### Tumour

The colon carcinoma cell line CC531, a 1,2-dimethylhydrazine-induced, moderately differentiated adenocarcinoma was used (Marquet *et al*, 1984). The cell line is transplantable in syngeneic WAG/RIJ rats. It exhibits a mutated K-ras gene in codon 12 (GGT to GAT), changing glycine to aspartic acid (unpublished data, laboratory of Dr R Koesters, DKFZ-Heidelberg, Germany). The tumour is weakly immunogenic, as determined by the immunization method of Prehn and Main (1957). The tumour can also be maintained in tissue culture. New tumour was produced from this culture by subcapsularly implantation in the liver. It is a relatively slowly progressing and poorly vascularized tumour. *In vivo* tumours were subsequently passaged serially.

### *In vitro* bioassay

CC531 cells were grown in RPMI 1640 (Gibco BRL, Paisley, UK) supplemented with 10% foetal calf serum (Harlan/Sera-Lab, UK), 1% penicillin (5000 IU ml^−1^), 1% streptomycin (5000 IU ml^−1^) and 1% L-glutamine (200 mM) (all Gibco BRL) in a humidified incubator at 37°C and 5% CO_2_. Before usage, the cells were trypsinized (1 min, 37°C), centrifuged (5 min, 700 g), resuspended and the viability measured by Trypan blue exclusion. Viability always exceeded 85%. For *in vitro* testing of proliferation inhibition, 1.0×10^4^ cells were seeded in flat-bottomed 96-well microtiter plates (Costar, USA). After 24 h the cells were incubated with different concentrations of the Y28 or empty construct for 48 h ranging from a multiplicity of infection (MOI) of 1–2.0×10^5^. Afterwards, cells were washed with PBS and fixed for 30 min with 10% trichloro-acetic acid at 4°C. Growth of tumour cells was measured using the sulpharhodamine-B assay according to the method of Skehan *et al* (1990). Tumour cell proliferation was measured using the formula: tumour growth=(test well/control)×100%. Five independent tests were performed for each point on the line.

### Animals

We used male inbred WAG/RIJ rats, weighing 250–300 *g*, obtained from Harlan-CPB (Austerlitz, The Netherlands). The rats were fed a standard laboratory diet. All animals were housed under standard conditions of light and accommodation. The protocol was approved by the committee for animal research of the Erasmus University, Rotterdam, The Netherlands. The experimental protocols adhered to the rules outlined in the Dutch Animal Experimentation Act of 1977 and the published Guidelines of the UKCCCR for the Welfare of Animals in Experimental Neoplasia (UKCCCR, 1998).

### *In vivo* colorectal liver metastases model

Following a standardized protocol, small viable CC531 tumour fragments of 1×2 mm were implanted under the liver capsule, one in the left and one in the right side of the left liver lobe, using a 19 G Luerlock needle. Experiments started at a tumour diameter of 5–6 mm, which was reached about 14 days after implantation of the tumour. When tumours reached a size of 25 mm in diameter or animals showed obvious signs of discomfort the animals were sacrificed.

### Isolated hepatic perfusion

**Figure 1:**
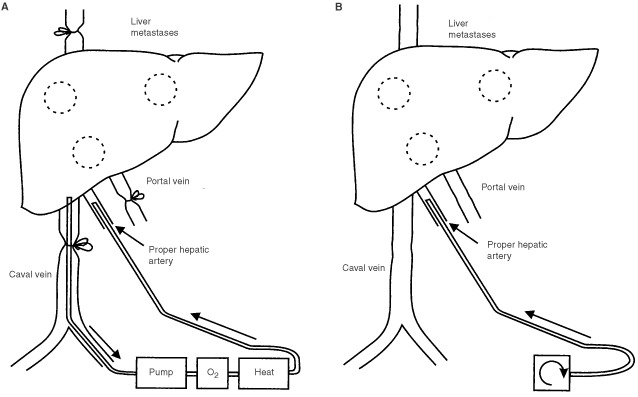
Schematic representation of (**A**) an isolated hepatic perfusion (IHP) and (**B**) a hepatic artery infusion (HAI).

This rat isolated liver perfusion model has been described in detail earlier by van IJken *et al* (2000). Briefly, the perfusion circuit consisted of an arterial inflow limb, a venous outflow limb and a collection reservoir/oxygenator (Figure 1A

). The circuit was primed with 10 ml Haemaccel (Behring Pharma, Amsterdam, The Netherlands). Arterial flow of 5 ml min^−1^ was maintained with a low-flow roller pump (Watson Marlow type 505 U, Falmouth, UK). Rats were perfused for 10 min with oxygenated Haemaccel and 1.0×10^11^ virus particles (v.p.), which was determined as the maximum tolerated dose (MTD), in a pilot study performed previously. Fifty IU of Heparin (Heparine Leo, The Netherlands) was added to the perfusate. The perfusate was oxygenated in the reservoir with a mixture of O_2_/CO_2_ (95% : 5%) and was kept at 38–39°C by means of a heat exchanger and a warm water bath. A temperature probe was positioned in the lumen of the arterial catheter, 5 cm away from the catheter tip. Afterwards, a wash out to remove all left viruses was performed by perfusing with 10 ml of oxygenated Haemaccel.

#### Surgical procedure of the isolated hepatic perfusion

Anaesthesia was induced and maintained with ether (Merck, Darmstadt, Germany). During the surgical procedure, with an average duration of 60–75 min, rats were kept at a constant temperature using a warmed mattress. A mid-line laparotomy was performed and the hepatic ligament exposed. The gastroduodenal side branch of the common hepatic artery was cannulated, positioning the tips of the cannula (0.025 inch outer diameter (OD), 0.012 inner diameter (ID), (Dow Corning, Michigan, USA)) in the proper hepatic artery. Through a small inguinal incision the femoral vein was exposed. To collect hepatic venous outflow a silicon cannula (0.047 inch OD, 0.025 ID), (Dow Corning, Michigan, USA) was introduced in the femoral vein and moved up into the caval vein positioning the tip of the cannula at the level of the hepatic veins.

Isolation of the hepatic vascular bed was obtained by temporarily ligating the common hepatic artery and the portal vein. The venous outflow limb was isolated by temporarily clamping the supra hepatic caval vein and by applying a temporary ligature around the infra-hepatic caval vein containing the cannula, cranial to the right adrenal vein. The mesenteric artery was temporarily clamped in order to reduce splanchnic blood pressure.

Following the procedure, the clamps on caval vein, portal vein, hepatic artery and mesenteric artery were released. The gastroduodenal artery and femoral vein were ligated and the gastroduodenal and femoral cannulas were removed.

### Single time and repeated hepatic artery infusion

#### Single time hepatic artery infusion

Anaesthesia was carried out with Hypnorm (Janssen Animal Health, Beerse, Belgium) and Ketamine (Apharmo B.V., Arnhem, The Netherlands). During the total procedure, which took on average 2 h and 20 min, rats were kept at a constant temperature with a warmed mattress and a heat producing light bulb. A mid-line laparotomy was performed and the hepatic ligament exposed. The gastroduodenal side branch of the common hepatic artery was then cannulated, positioning the tips of the cannula in the proper hepatic artery (Figure 1B). The cannula was connected to the infusion pump (B. Braun, Melsungen AG, Germany). 2.5×10^11^ v.p. (MTD) were dissolved in 1.5 ml of 0.9% NaCl/fractionated heparin solution (Fragmin, Pharmacia and Upjohn, Woerden, The Netherlands) (2500 IU Fragmin per 100 ml 0.9% NaCl). The viruses were infused in 1 h. Afterwards the cannula was flushed with 0.9% NaCl in order to infuse the remaining viruses in the cannula. During the infusion the arterial hepatic blood supply was maintained. At the end of the procedure the gastroduodenal artery was ligated and the cannula removed.

#### Repeated hepatic artery infusion

After positioning of the cannula in the gastroduodenal side branch of the hepatic artery as described above, the cannula was led through a flexible tube and connected via a bubble trap to a continuous 0.9% NaCl/Fragmin solution infusion pump which was set at a continuous infusion rate of 0.5 ml h^−1^. Treatment with 2.5×10^11^ v.p. (MTD) started at the day of operation and was repeated with 2.5×10^11^ v.p. every other day. The animals stayed in an adjusted filter top cage during the treatment schedule.

### Intravenous injection

Anaesthesia was induced and maintained with ether. A volume of 200 μl of PBS containing 2.5×10^11^ v.p. (MTD) was slowly injected into the penile vein using a syringe with a 25 G needle.

### Intratumoural injection

Anaesthesia was induced and maintained with ether. A mid-line laparotomy was performed and the tumours were exposed. Using a syringe with a 28 G needle, 5.0×10^10^ v.p. (MTD) in 50 μl sterile PBS were injected centrally in each tumour.

### *In vivo* transduction efficacy study

Experiments started at a diameter between 5 and 6 mm. In order to determine the transduction efficacy, tumour-bearing rats were treated with the AV1.0CMV.LacZ construct intravenously, by intratumoural injection, via a single time hepatic artery infusion, isolated hepatic perfusion and hepatic artery infusion repeated five times. PBS solution was used as a control. Twenty-four hours after treatment the animals were sacrificed. Tumours, liver and spleen were taken out, snap frozen in liquid nitrogen and stored at −80°C until further usage.

### X-Gal staining on cultured cells and cryosections

#### Staining of cultured cells

1.0×10^4^ CC531 cells were seeded in flat-bottomed 96-well microtiter plates. After 24 h the cells were incubated for 48 h with various concentrations of the LacZ construct ranging from a MOI of 1 up to 2.0×10^5^. Then, cells were washed with PBS and fixed for 30 min with 4% paraformaldehyde at 4°C. The cells were washed three times with PBS and stained with X-gal staining solution overnight at 37°C. This solution is a mixture of solution A: K_4_Fe(CN)_6_.3H_2_O 5 mM, K_3_Fe(CN)_6_ 5 mM in wash buffer (MgCl_2_ 2 mM, deoxycholate 0.01%, NP-40 0.02% in 0.1 M sodium phosphate buffer pH 7.8) and solution B: 5-bromo, 4-chloro, 3-indolyl β-d-galactopyranoside 50 mg ml^−1^ in dimethyl formamide) at ratio of 50 : 1. The cells were then washed once with PBS and stored at 4°C.

#### Staining of cryosections

Cryosections of snap frozen tissue samples were fixed in 4% paraformaldehyde for 30 min at 4°C. After three washes with phosphate buffered saline (PBS) pH 7.4, the sections were incubated overnight with X-gal staining solution at 37°C. Then, the sections were washed twice in PBS, counter-stained with haematoxylin, dehydrated with ethanol and xylene and coverslipped with entalan.

### Endothelial immunohistochemistry

In order to investigate transfected cells in relationship to the tumour vasculature cryosections were first stained by the X-gal method (see above) and secondly counter stained using a mouse-anti-rat antibody against rat endothelial cell antigen (RECA-1, Instruchemie, Hilversum, The Netherlands). After overnight X-gal staining sections were thoroughly rinsed with PBS. RECA-1 was diluted 1 : 10 in PBS and cryosections were incubated for 1 h. Thereafter sections were rinsed with PBS and incubated for 1 h with 1 : 100 diluted, in 5% normal rat serum in PBS, goat-anti-mouse peroxidase labelled antibody (DAKO, Carpenteria, CA, USA). After rinsing with PBS, positive cells were revealed by immunoperoxidase reaction with DAB-solution (DAB-kit, DAKO) and counter stained with haematoxylin.

### *In vivo* anti-tumour efficacy study

The various treatment modalities were started at a tumour size of 5–6 mm in diameter about 14 days after implantation of the tumour. After start of treatment tumour size was measured via a small midline laparotomy every fourth day. Tumour volume was calculated by using the following formula: tumour volume=A^2^×B×0.4. In which B is the largest diameter and A the diameter perpendicular to B, measured with a standardized calliper. In every treatment group, control rats were included. The AV1.0CMV construct was used as negative control in the efficacy study. In each treatment group, except for the 5×HAI treated rats, two animals were sacrificed 24 h after start of treatment in order to collect tumour and liver tissue. Tissues were snap frozen in liquid nitrogen and stored at −80°C until usage for immunohistochemistry.

### Y28 immunohistochemistry

Cryosections of snap frozen tissue samples were fixed in acetone for a few seconds. After three washes with PBS/Tween for 15 min the sections were incubated for 60 min with the 1 : 2000 diluted polyclonal rabbit anti-Y28 antibody (kindly provided by M Janicot, Institute Curie, Paris, France) at room temperature. After incubation the sections were washed twice in PBS/0.5% BSA, and incubated for 60 min with 1 : 20 diluted FITC-conjugated F(ab′)_2_ fragment of swine anti-rabbit immunoglobuline (Dako, Glostrup, Denmark). The sections were again washed with PBS/0.5% BSA. Afterwards slides were coverslipped with 90% PBS/glycerol and immediately analyzed and photographed with a Leica DM-RXA fluorescence microscope equipped with a Sony DXC950 digital camera.

### Toxicity study

In order to determine possible toxicity of the virus, rats were weighed every 4 days after start of treatment. Four days after treatment blood samples were taken via the tail vein. Serum was collected after centrifugation (14 000 r.p.m.) and stored at −80°C until further analysis. Liver functions (alkaline phosphatase, alaline aminotransferase, aspartate aminotransferase, total bilirubin and γ-glutamyl transpeptidase) and renal functions (creatinin and urea) were measured by spectophotometric analysis (ELAN-analyzer; Eppendorf-Merck, Hamburg, Germany). Thrombocyte, leukocyte, and erythrocyte numerations were also determined in these samples (Sysmex; Kyoto, Japan).

### Statistical analysis

*In vitro* and *in vivo* results were evaluated for statistical significance with the Kruskal–Wallis and Mann–Whitney *U*-tests with SPSS8.0 for Windows 98. A significance level of *P*<0.05 was used.

## RESULTS

### *In vitro* transfection efficacy and cytotoxicity study

Cells were incubated with different concentrations of the AV.1.0CMV.LacZ construct. After X-gal staining the percentage of transfected cells was calculated by scoring upon light microscopy. The concentration of virus required to achieve a 50% transfection rate was determined as TD 50 (Transfection Dose). Our estimated TD 50 in this experiment was determined at a MOI of 10 000. The maximum percentage of transfected cells was 64% at a MOI of 20 000. Above this concentration incubation of CC531 cells with LacZ virus resulted in cell death.

**Figure 2:**
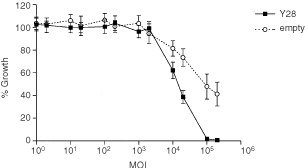
Growth of CC531 colon carcinoma cells *in vitro* after exposure to increasing concentrations of AV.1.0CMV.Y28 (Y28) or AV1.0CMV (empty). Five independent assays were performed in duplicate for each point on the line. Mean values (±s.e.m.) are shown.

Inhibition of proliferation of CC531 cells was observed with both constructs at a MOI higher than 2000. However, the Y28 construct showed a much stronger inhibition (Figure 2

). The MOI resulting in 50% growth inhibition (ID 50) was 15 000 for the Y28 construct and 89 000 for the empty construct. Therefore next to a direct cytotoxic effect of the adenovirus, an additive inhibitory effect of Y28 on CC531 cells *in vitro* was demonstrated.

### *In vivo* transduction efficacy study

**Figure 3:**
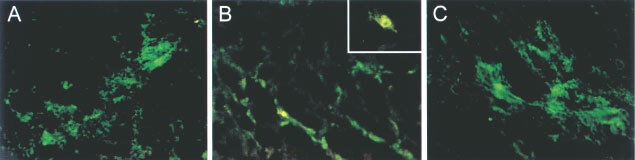
(**A**,**B**,**C**) Y28 fluorescence immunohistochemistry on cryosections of tumours collected 24 h after treatment *in vivo* with AV.1.0CMV.Y28 (Y28). (**A**) Tumour after IT, transfection around the needle track. (**B**) Foci of Y28 expression in tumour after IHP. (**C**) Expression in tumour after HAI. Original magnification: **A**, **B** and **C**: 16×, insert 40×. No staining was found in case of treatment with AV1.0CMV.

**Figure 4:**
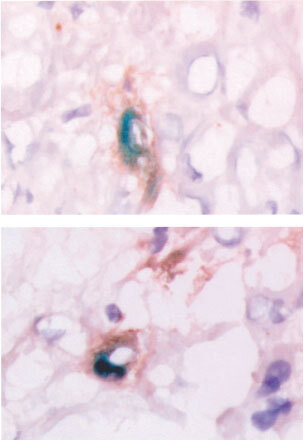
Two examples of X-gal stained and RECA stained cryosections of tumours of different animals after HAI treatment with AV.1.0CMV.LacZ. Tumour vessel (brown) and perivascular orientation of transfected cells (blue) are clearly visible. Original magnification: 40×. No staining was found in case of treatment with AV1.0CMV.

X-gal staining of cryosections and the Y28 immunohistochemistry studies revealed a needle track transfection pattern of tumour cells after intratumoural injection, with an estimated percentage of positive cells 5% (Figure 3A

). After IHP scattered foci of positive tumour cells per frozen section could be observed, 2–3% positive cells (Figure 3B). Almost no transfected tumour cells could be detected after single time intravenous or repeated (five times) intravenous or a single time hepatic artery infusion. After repeated HAI (5×HAI) however clear foci of transfection of tumour vasculature and peri-tumour vasculature transfection were observed, estimated 2–3% positive cells (Figures 3C and 4

*)*. Surprisingly, low transfection levels (±1%) were observed in liver tissue in all groups.

### *In vivo* anti-tumour efficacy study

In correspondence with the findings of the transduction efficacy study, intratumoural injection, intravenous injection, single time hepatic artery infusion and isolated hepatic perfusion with the Y28 construct did not result in any tumour response. All of the treated rats demonstrated progressive disease (Table 1

**Table 1:**
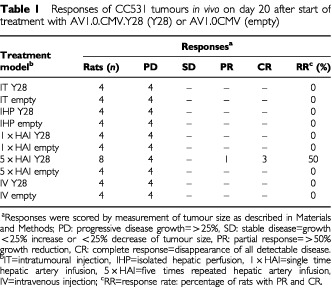
Responses of CC531 tumours *in vivo* on day 20 after start of treatment with AV1.0.CMV.Y28 (Y28) or AV1.0CMV (empty)

). Only the tumours injected IT with Y28 showed a slight, inhibition growth rate compared to empty construct. Strikingly, five times repeated hepatic artery infusion showed a complete response in three out of eight rats, a partial response in one rat and a growth inhibition in the other four tumours, resulting in an overall response rate of 50% (*P*<0.02 on day 12 and *P*<0.05 on day 16, compared to controls). All rats treated by 5×HAI with the empty construct demonstrated progressive disease (Figure 5

**Figure 5:**
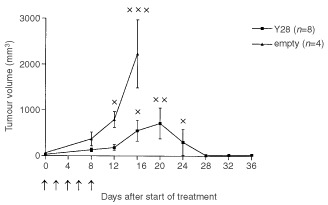
Tumour response of CC531 tumours *in vivo* after 5×HAI with AV.1.0CMV.Y28 (*n*=8) or AV1.0CMV (*n*=4) treated rats. On days 0, 2, 4, 6, and eight rats were infused (treatment schedule indicated by: ↑). Mean values (±s.e.m.) are shown (*P*<0.02 on day 12 and *P*<0.05 on day 16). At day 12–24 animals with progressive disease had to be sacrificed because of bulky tumour growth (indicated by: ×), so only the partial and complete responders are depicted after that time point.

). As a consequence of these results we performed five times repeated intravenous administration Y28 as an additive control group. This repeated treatment resulted in progressive disease in all animals (Table 1).

### Toxicity study

No severe hepatic, renal or hematological toxicity could be detected in any group after treatment with the Y28 or the empty construct. Sera from rats treated by repeated hepatic artery infusion were collected at day 8 after start of treatment. Levels of toxicity parameters measured in sera varied in a range of + and – 25% of the control values. The only abnormality observed was a doubling of γ-glutamyl transpeptidase (γGT) levels, which was detected in all groups and was equal after AV.1.0CMV.Y28 and AV.1.0CMV administration. Most changes in toxicity parameters seemed to be due to the surgical procedure or the viral constructs rather than the encoded gene. Haematological parameters showed a normal leukocyte count after IHP with Y28, but in all other groups there was a rise in leukocytes. The course of animal weights showed a decrease of 5%, except for IHP and 5×HAI treated rats, in which a transient weight loss of at maximum 10% was observed.

## DISCUSSION

For successful cancer gene therapy, tumour targeting is essential. This can be achieved by adjusting the vector, but also by adjusting vector delivery (Huard *et al*, 1995). In the present study, the aim was to target a poor vascularized colorectal tumour located in liver tissue and to study anti-tumour efficacy by targeting p21-ras. Delivery via intratumoural injection is an established method to achieve transfection but its clinical applicability is limited. We consider a transvascular approach a prerequisite for testing the real therapeutic window of gene therapy. IHP and HAI are transvascular administration modalities, which give rise to high local concentrations of the drug throughout the liver. de Wilt *et al* (2001) successfully performed isolated limb perfusion in sarcoma-bearing rats for the transfer of the IL-3β-gene. The IL-3β cytokine was excreted upon transfection of the cells and thereby induced a major bystander effect in this tumour model, which resulted in excellent tumour responses. The BN-175 tumour used in the IL-3β study is a high grade, rapidly growing tumour with an extensive vascularization. These characteristics offer superior possibilities for transfection. Fast growing tumours are more susceptible for genes driven by a CMV promoter (Ghazal *et al*, 1987). In contrast to the BN-175 tumour, CC531 tumours have a slow growth rate and are poorly vascularized. Apart from that, the immunologic activity of the liver, in comparison with a limb, is enormous.

*In vitro* transfection studies demonstrated that transfection of CC531 cells is possible. In a pilot study we investigated other cell lines as well and the transfection efficacy was equal compared to the colon carcinoma cell line used in this study. The *in vitro* cytotoxicity assay of AV.1.0CMV.Y28 construct showed effective tumour growth inhibition and cytotoxicity on CC531 cells. The TD 50 in the transfection efficacy study is about equal to the ID 50 of AV.1.0CMV.Y28 in the cytotoxicity assay. This strongly suggests that transfection of the ras mutated CC531 cells with Y28 anti-ras antibody results in proliferation inhibition. Next to this we performed *in vitro* bioassays on human umbilical vein derived endothelial cells (HUVECs) which do not harbour a ras mutation. On HUVECs AV.1.0CMV.Y28 hardly results in growth inhibition compared with the CC531 cell line (data not shown).

*In vivo* IT injection shows significant transfection of tumour cells on X-gal stained cryosections and immunohistochemistry. Strikingly, a single administration of AV.1.0CMV.Y28 intratumourally did not result in a significant tumour response. This can be explained by the fact that IT injection results in transfection around the needle track only. A trans-endothelial route causes a more homogeneous distribution of transfection of the tumour. We have reported this previously in the rat sarcoma model particularly at the viable rim of the tumour (de Roos *et al*, 2000).

Bilbao *et al* (2000) found that a blood–tumour barrier in hepato-cellular carcinoma in rats limits the gene transfer in tumours greater than 5 mm in diameter. Moreover, they also concluded that tumours between 2 and 5 mm could only be transfected by hepatic artery infusion. As already mentioned we started treatment at a tumour diameter of about 5 mm, so a blood–tumour barrier may well play a role in the limited transvascular transfection we observed. After multiple HAI, the transfection rate of tumour tissue still remains low, however in the AV1.0CMV.LacZ and Y28 immunohistochemistry experiments transfection could be demonstrated around tumour vessels.

It is known that down regulation of p21-ras causes a decrease of vascular endothelial growth factor (VEGF) production (Arbiser *et al*, 1997; Okada *et al*, 1998; Zhang *et al*, 2001). A bystander effect caused by down regulation of VEGF after p53-gene therapy *in vivo* has already been described (Bouvet *et al*, 1998). Expression of the Y28 construct may cause an anti-angiogenic effect by this ras-VEGF-pathway. A higher transfection of perivascular tumour cells and, as a consequence of this, a reduction of VEGF levels around tumour vessels upon 5×HAI could play a role in the anti tumour effect we observed.

Our experiments indicate that indirect immunological anti-tumour effect by repeated adenovirus administration can be ruled out, because not only five times repeated HAI with the empty control vector, but also five times repeated intravenous administration with AV1.0.CMV.Y28 did not result in any anti-tumour efficacy. We conclude that it is a prerequisite to deliver this vector loco-regionally and in a repetitive way.

Targeted gene therapy is a major issue in the development of gene therapy towards clinical trials. Furthermore, gene therapy has to be safe. In this study we report on a successful repeated administration of adenoviral vectors without significant toxicity. We demonstrated that loco-regional gene therapy of slowly progressing, poorly vascularized colon carcinoma liver metastases is feasible and that repeated treatment might offer possibilities for future gene therapy trials.
